# Aleutian Mink Disease Virus and Humans

**DOI:** 10.3201/eid1512.090514

**Published:** 2009-12

**Authors:** Jørgen R. Jepsen, Francesco d’Amore, Ulrik Baandrup, Michael Roost Clausen, Elisabeth Gottschalck, Bent Aasted

**Affiliations:** Hospital of South-Western Denmark, Esbjerg, Denmark (J.R. Jepsen); Aarhus University Hospital, Aarhus, Denmark (F. d’Amore, M.R. Clausen); Aalborg University, Aalborg, Denmark (U. Baandrup); Gl. Ringstedvej 63 D, Holbæk, Denmark (E. Gottschalck); University of Copenhagen, Copenhagen, Denmark (B. Aasted)

**Keywords:** Parvoviridae, blood-borne pathogens, zoonoses, viruses, dispatch

## Abstract

Reports of a possible relationship between Aleutian mink disease parvovirus (AMDV) and human infection are rare. However, 2 mink farmers with vascular disease and microangiopathy similar to that in mink with Aleutian disease were found to have AMDV-specific antibodies and AMDV DNA. These findings raise the suspicion that AMDV may play a role in human disease.

Autonomous parvoviruses, such as Aleutian mink disease virus (AMDV), cause a broad spectrum of diseases in animals and man. Acute disease manifests itself as a lytic infection of rapidly dividing cells; chronic disease reflects a restricted or abortive infection of specific cell types (*1*). Aleutian disease (AD) is known to produce clinical signs in mink and ferrets only (*2*,*3*), although other mammals have reportedly been antibody positive.

In adult mink, AD is a persistent, slowly progressive AMDV infection in which a dysregulated immune system and a postinfectious antibody response cause an immune complex–mediated vasculitis (*2*). Perivascular and glomerular immune complexes (*2*,*4*,*5*) can cause membranoproliferative glomerulonephritis (*6*) and segmental or circumferential arteritis (*4*) with mononuclear infiltration, fibrinoid necrosis and deposits, and increased intimal cellularity. Mononuclear cells may surround the vessel, and connective tissue proliferation and necrosis in the tunica elastica media narrow the lumen (*7*). In mink kits, AD causes an acute cytopathic infection of alveolar cells, which leads to respiratory distress and death (*8*).

Reports of a possible relationship between AMDV and human infection are rare (*9*). Histopathologic features like those in AMDV-infected mink have been described for 2 patients in the early 1960s (*10*). Exposed laboratory workers have had persistent anti-AMDV antibodies for up to 18 months; however, injection of their antibody-positive blood into Aleutian mink caused neither lesions nor AMDV-antibody production (*11*). In vitro studies have demonstrated a permissive infection (production of infectious progeny) of human macrophages with the Utah I strain of AMDV (*12*). We report finding anti-AMDV antibodies and AMDV genome in tissue from 2 mink farmers with relevant virus exposure and clinical disease similar to that in mink with AD.

## The Study

We examined AMDV antibody from each of the 2 patients by countercurrent and line electrophoresis (*13*). AMDV DNA was identified by standard and nested PCR. DNA was extracted from lymph nodes (patient 1) and from peripheral blood and bone marrow (patient 2) before amplification with AMDV-specific primers. AMDV DNA was identified by 2 different sets of primers in the standard PCR (5–600 bp) and with 2 complete different internal primers in the nested PCR (200 bp). PCR products were cloned, and some clones were sequenced to confirm the presence of AMDV DNA. All PCR reactions were done with appropriate controls.

**Figure 1:**
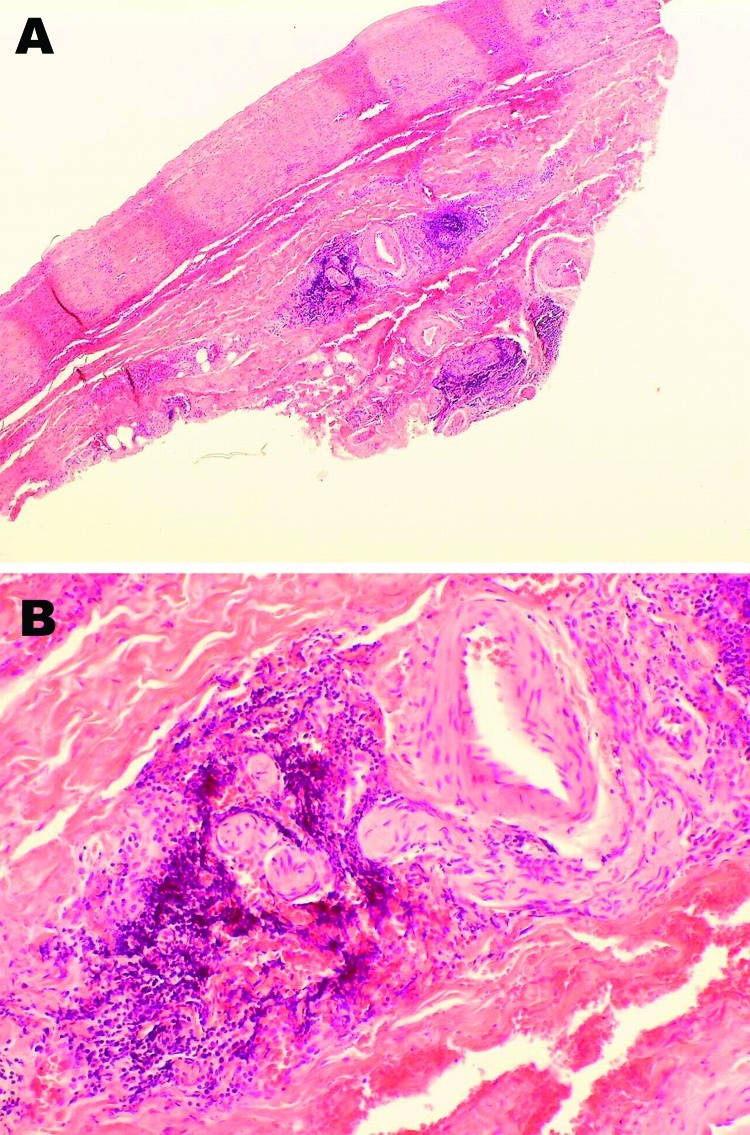
Histopathologic appearance of abdominal aortic biopsy sample from 35-year-old mink farmer in Denmark who had been exposed to Aleutian mink disease parvovirus−infected mink for 10 years (patient 1). A) Perivascular, adventitial lymphoplasmacytoid infiltration. Original magnification ×4. B) Minimal atherosclerotic changes. Original magnification ×20.

Patient 1 was a mink farmer who had been exposed to AMDV-infected mink for 10 years. When he was 22 years of age, toe ulceration and claudication developed. Arteriography showed bilateral occlusions of several lower limb arteries and associated development of a collateral network of vessels. At the age of 25, he underwent embolectomy, and the removed tissue showed vessel wall inflammation with a granulomatous appearance but no necrotizing lesions or epitheloid or eosinophilic infiltration. Over the next 10 years, despite surgical attempts to revascularize and treatment with anticoagulant drugs, his condition deteriorated: his renal, mesenteric, and axillary arteries became stenosed, and his right leg was amputated. Antibodies to AMDV were found in his serum at the end of these 10 years and at 2 subsequent measurements after 1 additional year. An abdominal aortic biopsy showed adventitial lymphoplasmacytic cell infiltration and minimal atherosclerosis (Figure 1). A lymph node biopsy sample showed modest reactive changes and T-zone hyperplasia, and AMDV DNA was identified in the sample. At 35 years of age, the patient had a positive serologic result for anti-AMDV antibodies and severe claudication. Subsequent testing 1 and 2 years later showed negative results for AMDV antibodies and AMDV genome. The patient died in 1999, at 40 years of age, at which time his clinical condition resembled that of bilateral pneumonia. No specific infectious agent was identified. Postmortem examination showed periarterial collagen deposits, adventitial focal mononuclear accumulations, neutrophil infiltration in the media, fibrosis-related hyperplasia, lipid deposition and calcifications of the intima, and microabscesses within intraluminal thrombotic material.

**Figure 2:**
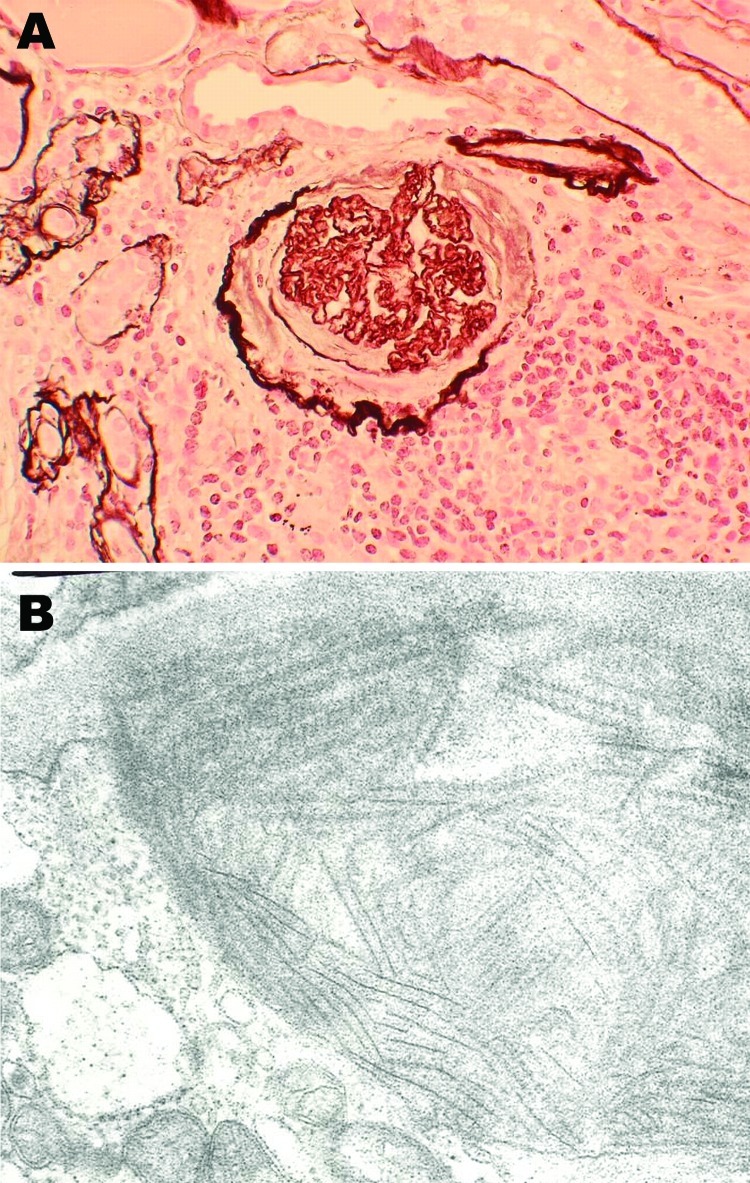
Histopathologic appearance of renal biopsy sample from 54-year-old mink farmer in Denmark who had been exposed to Aleutian mink disease parvovirus−infected mink for 34 years (patient 2). A) Glomeruli with hypercellularity and crescents. Original magnification ×20. B) Electron microscopy showing distinct extracellular deposits of coarse 20-nm fibrils (microtubular structures) characterized as immunotactoid glomerulopathy. Original magnification ×100,000.

Patient 2 was also a mink farmer. He had been exposed to AMDV since the age of 20. At 54 years of age, 2 years after an extensive outbreak of AMDV among his mink, he was hospitalized for chronic glomerulonephritis. A renal biopsy sample showed endocapillary and mesangial proliferative glomerulonephritis with abundant focal semilunes (Figure 2, panel A). Immunofluorescence showed anti-immunoglobulin M and anti-C3 antibodies localized to the renal capillaries. Electron microscopy showed organized fibrillar deposits of stacked microtubules of ≈20 nm (Figure 2, panel B), consistent with fibrillary glomerulonephritis (*14*), an idiopathic condition characterized by polyclonal immune deposits with restricted gamma isotypes. No seroimmunologic information was available for the patient at this time. Immunosuppression improved his renal function, and he remained stable while receiving continuous immunosuppressive medication.

Eight years later, patient 2 was readmitted to the hospital for diarrhea, vomiting, pyrexia, asthenia, and confusion. Cerebrospinal fluid contained high levels of protein and pleocytosis, but no specific infectious agent could be isolated from the cerebrospinal fluid or blood. Magnetic resonance imaging showed an increased meningeal signal over both cerebral hemispheres. Subsequent investigations repeatedly demonstrated anti-AMDV antibodies and AMDV DNA in peripheral blood and bone marrow. Serum was still positive for AMDV antibodies 2 years later. Despite treatment with antimicrobial drugs, the patient further deteriorated and died in 2004, at age 63, after an additional year of hospitalization. At postmortem examination, the kidneys were reduced in size with evidence of cortical attenuation. Increased mesangial hypercellularity was observed. An adenocarcinoma of the right lung had metastasized to the suprarenal glands, liver, and mesenterium. Coronary arteries and the aorta were moderately atherosclerotic.

## Conclusions

The clinical history, histopathologic features, and molecular findings for the 2 mink farmers exposed to AMDV were similar to those described for AD in mink. The combination of clinical and laboratory findings is unique for these patients compared with previous reports. These 2 patients had micro- and macroangiopathic lesions and prolonged persistence of serum antibodies to AMDV and AMDV DNA.

On the basis of its early onset, dissemination, and severity, the slight atherosclerosis found in some histopatologic specimens from patient 1 represent a consequence of the pre-existing arteritis rather than a primary condition. Buerger disease is unlikely on the basis of cytopathologic and histopathologic findings, and other vasculitic disorders were excluded on the basis of serologic findings. The arteritis was similar to the autoimmune vascular lesions accompanying AD in mink with adventitial lymphocytic infiltration (*4*). The histopathologic findings for patient 2, in whom the autoimmune glomerulonephritis was diagnosed 8 years before the first measurement of anti-AMDV antibodies, resembled the immune complex–mediated glomerulonephritis in mink with AD (*6*).

For patient 1, anti-AMDV antibodies persisted 4 years from his last exposure to mink, exceeding the longest reported duration of positive AMDV response in a human in the absence of virus exposure (*11*). Similarly, patient 2 had a long-lasting antibody response, although under potentially persisting exposure. The persistence of anti-AMDV antibodies in patient 2 may reflect host-related factors in the modulation of immune response to chronic antigen stimulation. In mink, host-related factors influence their susceptibility to AMDV infection and correlate with clinical manifestations from an asymptomatic condition to overt disease. One may speculate whether the overrepresentation of malignant lymphoma in mink farmers could reflect a part of a disease spectrum paralleling monoclonal plasma cell proliferation in mink (*15*).

That AMDV DNA was found only in the first biopsy sample from patient 1 may weaken the hypothesis of virus replication. However, it may reflect technical difficulties with DNA amplification after paraffin embedding of the specimen or a possible later clearance of the virus from infected tissues. Regardless, we have described our clinicopathologic and molecular findings with the goal of raising awareness about the possible role of AMDV replication in human disease.
